# Sarconesin: *Sarconesiopsis magellanica* Blowfly Larval Excretions and Secretions With Antibacterial Properties

**DOI:** 10.3389/fmicb.2018.02249

**Published:** 2018-09-28

**Authors:** Andrea Díaz-Roa, Manuel A. Patarroyo, Felio J. Bello, Pedro I. Da Silva

**Affiliations:** ^1^Laboratório Especial de Toxinologia Aplicada, Instituto Butantan, São Paulo, Brazil; ^2^PhD Programme in Biomedical and Biological Sciences, Universidad del Rosario, Bogotá, Colombia; ^3^Biomedical Sciences Institute, Universidade de São Paulo, São Paulo, Brazil; ^4^Molecular Biology and Immunology Department, Fundación Instituto de Inmunología de Colombia, Bogotá, Colombia; ^5^Basic Sciences Department, School of Medicine and Health Sciences, Universidad del Rosario, Bogotá, Colombia; ^6^Faculty of Agricultural and Livestock Sciences, Program of Veterinary Medicine, Universidad de La Salle, Bogotá, Colombia; ^7^Medicine Faculty, Universidad Antonio Nariño, Bogotá, Colombia

**Keywords:** antimicrobial peptide, *Sarconesiopsis magellanica*, sarconesin, larval therapy, insect peptide

## Abstract

Larval therapy (LT) is an alternative treatment for healing chronic wounds; its action is based on debridement, the removal of bacteria, and stimulating granulation tissue. The most important mechanism when using LT for combating infection depends on larval excretions and secretions (ES). Larvae are protected against infection by a spectrum of antimicrobial peptides (AMPs); special interest in AMPs has also risen regarding understanding their role in wound healing since they degrade necrotic tissue and kill different bacteria during LT. *Sarconesiopsis magellanica* (Diptera: Calliphoridae) is a promising medically-important necrophagous fly. This article reports a small AMP being isolated from *S. magellanica* ES products for the first time; these products were obtained from third-instar larvae taken from a previously-established colony. ES were fractionated by RP-HPLC using C18 columns for the first analysis; the products were then lyophilised and their antimicrobial activity was characterized by incubation with different bacterial strains. These fractions’ primary sequences were determined by mass spectrometry and *de novo* sequencing; five AMPs were obtained, the Sarconesin fraction was characterized and antibacterial activity was tested in different concentrations with minimum inhibitory concentrations starting at 1.2 μM. Potent inhibitory activity was shown against Gram-negative (*Escherichia coli* D31, *E. coli* DH5α, *Salmonella enterica* ATCC 13314, *Pseudomonas aeruginosa* 27853) and Gram-positive (*Staphylococcus aureus* ATCC 29213, *S. epidermidis* ATCC 12228, *Micrococcus luteus* A270) bacteria. Sarconesin has a significant similarity with Rho-family GTPases which are important in organelle development, cytoskeletal dynamics, cell movement, and wound repair. The data reported here indicated that Sarconesin could be an alternative candidate for use in therapeutics against Gram-negative and Gram-positive bacterial infections. Our study describes one peptide responsible for antibacterial activity when LT is being used. The results shown here support carrying out further experiments aimed at validating *S. magellanica* AMPs as novel resources for combating antibacterial resistance.

## Introduction

Larval therapy (LT) involves applying sterile larvae (usually Diptera from the Calliphoridae family) to an infected non-healing wound (Raposio et al., 2017); its action is based on four mechanisms: removing necrotic tissue (debridement), disinfecting microorganisms, including methicillin-resistant *Staphylococcus aureus* (MRSA) (Robinson, 1935; Mumcuoglu, 2001; Bexfield et al., 2004; Nigam et al., 2010), inhibiting and eradicating biofilms (van der Plas et al., 2008; Cazander et al., 2009; Gottrup and Jorgensen, 2011) and stimulating granulation tissue for enhancing healing (Church, 1996; Thomas A.M. et al., 1999; Sherman et al., 2000; Mumcuoglu, 2001; Wolff and Hansson, 2005; Spilsbury et al., 2008).

It has been proposed that larvae release antimicrobial ingredients into wounds in response to infection; some of these ingredients are low molecular weight bacteriostatic compounds, such as p-hydroxybenzoic acid, p-hydroxyphenylacetic acid, dioxopiperazine proline (Huberman et al., 2007) and an enigmatic compound (C_10_H_16_N_6_O_9_) known as seraticin (Nigam et al., 2010). Other compounds are antimicrobial peptides (AMPs) originating from the immune system that, when applied into wounds, contribute to their healing (Thomas A.M. et al., 1999; Bexfield et al., 2004). Such insect peptides belong to the diptericin, cecropin, and defensin groups (Hoffmann and Hetru, 1992; Bulet and Stocklin, 2005). Lucifensin is one of the well characterized AMPs; it is derived from *Lucilia sericata* larvae and has been found as a constituent of larval excretions and secretions (ES) (Cerovsky et al., 2010). This molecule was originally isolated from *Lucilia sericata* larval intestine, later being detected in the salivary glands, fat body and haemolymph. However, it has been shown that it is the larval immune system which induces the production of these substances in the fat body when activated in response to an infectious environment (Valachova et al., 2013) for its rapid release into the haemolymph.

Insects respond to bacterial attacks by rapidly producing AMPs which have a broad spectrum of activity against Gram-positive and Gram-negative bacteria, and against fungi; more recently, it has been demonstrated that AMPs have activity against some parasites and viruses (Yi et al., 2014). Insect-isolated AMPs can be classified on the basis of their sequence and structural characteristics into three categories: linear peptides which can form an alpha-helical structure and contain no cysteine residues, such as cecropins; cyclic peptides containing disulphide bridges, such as defensins; and linear peptides having remarkable contents of one or two amino acid (aa) residues, mostly proline and/or glycine (e.g., pyrocoricins and diptericins) (Bulet et al., 1999).

These peptides are conserved host immune system evolutionary components forming part of the first line of defense against infections and have been identified in almost all life forms. Insect isolates make up the most abundant group of more than 2,798 AMPs listed in the antimicrobial peptide database^1^. AMPs are synthesized in the fat body (the equivalent of the liver in mammals), epithelial cells and certain haemolymph cells (the equivalent of mammalian blood), and spread throughout the body by this medium for counteracting infection. Most of these peptides are cationic AMPs having a molecular mass of less than 5 kDa (Brown and Hancock, 2006). In contrast to conventional antibiotics, AMPs do not induce microbial resistance and require only a short time to induce microorganism death (Yeaman and Yount, 2003).

Microorganisms’ resistance to antibiotics represents an ever-increasing difficulty; such situation becomes even more relevant in relation to chronic wounds which are difficult to heal in patients suffering underlying disease (such as diabetes and cardiovascular failure). Polymicrobial colonization by different bacterial strains often occurs in this type of lesion, forming a biofilm, thereby making them more difficult to treat, control and/or eradicate. Recent studies (O’Meara et al., 2014) have demonstrated that conventional antibiotics do not promote chronic wound healing, in addition to generating resistance in bacteria, which is why their general use has been questioned for treating the bacteria colonizing this type of wound. There is thus a need to introduce new or re-emerging strategies which may be effective against microorganisms in chronic, necrotic and infected wounds which do not respond to antibiotic therapy.

Identifying and characterizing antibacterial compounds involved in larval ES during LT is the starting point for the search and typing of natural molecules in insects, mainly dipterans from the Calliphoridae family (Yeaman and Yount, 2003). AMPs have been isolated from purified larvae in just *L. sericata* (Cerovsky et al., 2010), *L. cuprina* (El Shazely et al., 2013), and *Calliphora vicina* (Chernysh et al., 2015) whilst lucifensins have been isolated from larvae, purified, characterized and evaluated. They have been shown to be mainly effective against Gram-positive bacteria, such as MRSA and its strains (Andersen et al., 2010;Cerovsky and Bem, 2014). Amongst the most recent studies on antimicrobial peptides isolated from flies from the Calliphoridae family different to those obtained from larval ES, it is worth mentioning a study by Yakovlev et al. (2017) (Yakovlev et al., 2017), who studied the presence of AMPs in the culture medium from both fat bodies and haemocytes derived of *Calliphora vicina* larvae. They demonstrated that both cell types synthesized and released an AMPs complex to the culture medium, corresponding to defensins, cecropins, diptericins, and proline-rich peptides. In another study, AMPs extracted from *C. vicina* larval haemolymph were applied in environments extremely contaminated by germs forming biofilms (under *in situ* and *in vitro* conditions), exhibiting strong destructive activity of the matrix and of bacteria adhered to it, these bacteria were resistant to conventional antibiotics, such as *Escherichia coli, Staphylococcus aureus*, and *Acinetobacter baumannii* (Gordya et al., 2017). Likewise, this AMP complex containing a combination of defensins, cecropins, diptericins and proline-rich peptides, and interacting synergistically with antibiotics of various classes, produced a much stronger action on the bacterial strains (*Staphylococcus aureus, Escherichia coli, Pseudomonas aeruginosa, Klebsiella pneumoniae*, and *Acinetobacter baumannii*) and the biofilm materials, when compared with the antibacterial effect on the same strains in a model of planktonic cultures (Chernysh et al., 2018).

The *S. magellanica* species has been reported in Argentina (Mariluis and Mulieri, 2003), Bolivia, Chile, Ecuador and Perú (Pape et al., 2004); Mariluis and Peris have described it as being present in areas over 900 masl (Mariluis and Peris, 1984). In Colombia, it is found in Boyacá and Cundinamarca departments (Hardy, 1966). *S. magellanica* was reported as the first species colonizing decomposing pigs in Bogotá (an animal bio-model similar to that of humans) (Goff, 2001). Its antibacterial activity has been confirmed, giving better results than those for *L. sericata* (Diaz-Roa et al., 2014) and it has already been evaluated regarding LT, leading to good effects concerning diabetic wound cicatrisation (Cruz-Saavedra et al., 2016) and in *Leishmania* lesions (Cruz-Saavedra et al., 2016).

This study was thus aimed at characterizing a novel AMP purified from *S. magellanica* ES. The antimicrobial activity of the peptide so obtained against various Gram-positive and Gram-negative bacteria was also evaluated.

## Materials and Methods

### Adult *S. magellanica* Capture and Colony Maintenance

Insect capture and colony maintenance followed a previously described procedure (Diaz-Roa et al., 2014). Adult *S. magellanica* forms were captured in the mountainous part of Bogotá’s Parque Nacional; the park is located 2,600 masl (4°37′8.90N; 74°3′27.73W). Entomological nets were used for collecting insects which were then stored alive in glass flasks for being transported to the Universidad del Rosario’s Medical and Forensic Entomology laboratory. The specimens were kept in 45 × 45 × 45 cm Gerber cages at 20–25°C, with 60–70% relative humidity and a 12/12 h photoperiod. The adult forms were fed on a sugar solution (carbohydrate source) and pigs’ liver as protein feed necessary for providing continuity for the biological cycle (Rueda et al., 2010); after adults had laid eggs on the liver they were placed in a glass flask with a liver slice until maggots hatched. The maggots were kept in this flask throughout the 3 instars until they reached the pre-pupa stage; they were then put in a flask containing sand until the adults emerged to be released in the same cages to continue the cycle. Third instar maggots were used for extracting their ES.

### Extracting *S. magellanica* ES

*S. magellanica-*derived ES were collected from third instar larvae, following a previously described procedure (van der Plas et al., 2008); about 200 larvae were used in each assay. Third-instar larvae were incubated with a bacterial suspension (OD595 = 0.5) of each selected strain to activate the immune system and enhance the expression of products having antibacterial activity (Kawabata et al., 2010; Jiang et al., 2012). They were then placed in a 15 mL Falcon tube and disinfected by adding 0.5% formaldehyde for 5 min followed by replacing this solution with 0.5% hypochlorite with constant shaking for the same amount of time and washed with sterile PBS; 2 mL sterile PBS was then added to the larvae which were incubated at 25°C for 1 h. The larval ES mixture was removed by using a syringe and placed in another tube to continue centrifuging at 13,000 g at 4°C for 10 min. The precipitate was discarded and the supernatant with the ES was sterilized by filtering through a 0.22 μm membrane and stored at -70°C.

### Peptide Purification

ES were partially purified by Sep-Pak C18 disposable columns for the first analysis; bound material was eluted with 80% acetonitrile (ACN) in acidified water and freeze-dried. *S. magellanica* hydrophobic ES (80%) were then lyophilised and reconstituted in 2mL trifluoracetic acid (0.05% TFA). ES were purified by semi-preparative reverse-phase high-performance liquid chromatography (RP-HPLC) using a C18 Jupiter column (10 μm; 300A; 10mm × 250mm) at 2mL/min flow rate, as previously described (Hou et al., 2011). Fractions were collected manually, absorbance being monitored at 225nm. Each fraction’s antibacterial activity was then determined. RP-HPLC (1mL/min flow rate) was then used with fractions having antibacterial activity, using an analytical C18 Jupiter column (10 μm; 300A; 4,6mm × 250mm). The Sarconesin gradient was open, ACN concentration ranging from 44% to 54%. Absorbance was monitored at 225nm, fractions were collected manually and antibacterial activity was tested.

### Antimicrobial Assays

A liquid growth inhibition assay was used for evaluating the fractions’ antibacterial activity (Bulet, 2008; Wiegand et al., 2008). Lyophilised fractions were suspended in 500 μL Mili Q water; the assay was carried out using 96-well sterile plates. 20 μL of the fractions were aliquoted into each well with 80 μL of the bacterial dilution, to 100 μL final volume. Bacteria were cultured in poor nutrient broth (PB) (1.0 g peptone in 100 mL of water containing 86 mM NaCl at pH 7.4; 217 mOsm). Exponential growth phase cultures were diluted to 5 × 10^4^ CFU/mL (DO = 0.001) final concentration (Hetru and Bulet, 1997; Bulet, 2008; Poppel et al., 2015). Sterile water and PB were used as growth control, and streptomycin was used as growth inhibition control. Microtitre plates were incubated for 18 h at 30°C; growth inhibition was determined by measuring absorbance at 595 nm. The assay for determining the minimum concentration of peptide required to achieve 100% growth inhibition was performed using a serial dilution in 96-well sterile plates at 100 μL final volume (Silva et al., 2000;Lorenzini et al., 2003;Riciluca et al., 2012); 20 μL stock solution was used in each microtitre plate well at twofold serial dilution and added to 80 μL of the bacterial dilution. The strains used were *Staphylococcus aureus* ATCC 29213, *S. epidermidis* ATCC 12228, *Escherichia coli* D31, *E. coli* DH5α, *Pseudomonas aeruginosa* 27853, *Salmonella enterica* ATCC 13314, and *Micrococcus luteus* A270. Microbial growth was measured by monitoring optical density at 595 nm and assays were performed in triplicate (PerkinElmer Victor 3TM 1420 multilabel counter). The bacterial growth curve of *S. aureus* with Sarconesin MIC and ½ MIC was measured every 15 min for 1 h and then every hour for 12 h. Graph was background-corrected by subtracting the OD595 of medium without bacteria (Velema et al., 2013; Magi et al., 2015).

### Cytotoxicity (CC)

The toxicity of Sarconesin peptide against VERO cells (African green monkey kidney fibroblast) was evaluated. Cells were obtained from the American Type Culture Collection (ATCC CCL81; Manassas, VA, United States) and maintained in DMEM culture medium, supplemented with 10% heat-inactivated calf serum. CC was determined using the MTT colorimetric assay. Briefly, the cells were seeded in 96-well plates (2 × 10^5^ cells/well) and cultured for 24 h at 37°C in a humidified atmosphere containing 5% CO_2_. Eight two-fold serial dilutions of peptide were performed with DMEM to give solutions with final concentrations ranging from 4, 7 to 600 μM. Varying concentrations were added and allowed to react with the cells for 48 h, followed by the addition of 20 μL MTT (5 mg/mL in PBS) for another 4 h at 37°C. Formazan crystals were dissolved by adding 150 μL isopropanol and incubating at room temperature until all crystals were dissolved. Absorbance at 550 nm was measured using a microplate ELISA reader. Cell survival was calculated using the following formula: survival (%) = (A550 of peptide-treated cells/A550 of peptide-untreated cells)^∗^100 (Sayegh et al., 2016).

### Haemolytic Activity

Fresh human red blood cells (hRBCs) were washed 3 times with PBS (35 mM phosphate buffer, 0.15 M NaCl, pH 7.4) by centrifugation for 7 min at 1000 × g, and resuspended in PBS to a final 4% (v/v) concentration. Sarconesin solutions (serial 2-fold dilutions in PBS) were added to 100 uL hRBC suspension to a final 200 μL volume, and incubated for 1 h at 37°C. Hemoglobin release was monitored by measuring the supernatant absorbance at 405 nm with a Microplate ELISA Reader. The haemolysis percentage was expressed in relation to a 100% lysis control (erythrocytes incubated with 0.1% Triton X-100); PBS was used as a negative control (Nan et al., 2012;Chaparro and da Silva, 2016).

### Mass Spectrometry

Active antibacterial fractions were analyzed by mass spectrometry LC-MS/MS on a LTQ-Orbitrap Velos (Thermo Scientific) coupled to an Easy-nLCII liquid nano-chromatography system (Thermo Scientific). The chromatographic step involved using 5 μL of each sample automatically on a C18 pre-column (100 μm I.D. × 50 mm; Jupiter 10 μm, Phenomenex Inc., Torrance, California, United States) coupled to a C18 analytical column (75 μm I.D. × 100 mm; ACQUA 5 μm, Phenomenex Inc.). The eluate was electro-sprayed at 2 kV and 200°C in positive ion mode. Mass spectra were acquired by FTMS analyser; full scan (MS1) involved using 200–2,000 m/z (60,000 resolution at 400 m/z) as mass scan interval with the instrument operated in data dependent acquisition mode, the five most intense ions per scan being selected for fragmentation by collision induced dissociation. The minimum threshold for selecting an ion for a fragmentation event (MS2) was set to 5,000 cps. The dynamic exclusion time used was 15 s, repeating at 30 s intervals.

### Bioinformatics

The MS/MS peak list files were submitted to an in-house version of the MASCOT server (Matrix Science, United States) and screened against the Uniprot database. PEAKS 8.5 (Bioinformatics Solutions Inc., Waterloo, Ontario, Canada) *de novo* sequencing/database search software was used for establishing sequences. Analysis involved 10 ppm error tolerance for precursor ions and 0.6 Da for fragment ions. Oxidation was considered a variable modification.

The Sarconesin sequence was analyzed for similarities with the *L. sericata* and *L. cuprin*a genome and transcriptome and also with other proteins registered in the National Center for Biotechnology (NCBI) public database, using the Basic Local Alignment Search Tool (BLASTp), with default parameters^2^ (Altschul et al., 1997). The sequences’ physical-chemical parameters were calculated using the PepCalc tool^3^. Gene Runner was used for nucleotide translation to protein and Seaview (Gouy et al., 2010) and Boxshade^4^ was used for making and formatting alignments’ shaded background. The Chimera structure prediction tool (accessed through the European Bioinformatics Institute^5^ was used for obtaining the three-dimensional (3D) images of secondary structure.

### Circular Dichroism (CD)

The far-UV (190–250nm) CD spectrum of the peptide was recorded in a Jasco J810 spectropolarimeter (Jasco Inc., Japan) at 25°C and in a 0.1cm path length quartz cell. All CD spectra were recorded after accumulation of 4 runs and smoothed using a FFT (Fast Fourier Transform) filter to minimize background effects. The solvents used in the experiment were pure water and 10, 30 and 50% v/v solutions of 2,2,2 trifluoroethanol (TFE) in water.

### Mechanism of Action

#### Membrane Integrity and Esterase Activity

Mid-log phase *E. coli* cells (2 × 10^8^ CFU/mL) were incubated with or without MIC peptide solution at 37°C. The bacterial membrane integrity was measured by fluorometry and microscopy using propidium iodide (PI) to 60 μM final concentration in the dark for 15 min, followed by measuring fluorescence with excitation/emission wavelengths of 485/620 nm (Faisal et al., 2016). For Esterase activity, 180 μL were transferred to a 96-well black plate which was added 20 μL of 250 μM 5(6)-Carboxyfluorescein diacetate (CFDA), incubated in dark for 30 min, followed by measurement of fluorescence with excitation/emission wavelengths of 485/535 nm (Nocker et al., 2011; Yang et al., 2017). PI microscope slides, were made by depositing drops of melted agarose 1% (w/v); after placing 20 μL of the cells onto solidified agar pad for immobilisation, the dried culture was covered with a glass coverslip and observed under a microscope (Carretero et al., 2018). Microscopy was performed using a Leica TCS SP8 confocal laser scanning microscope, the images were processed with Leica software LAS X.

#### DNA Staining

Treated and untreated bacterial cells were fixed on a slide, permeabilised with ethanol, and stained with 4’,6-diamidino-2-phenylindole (DAPI) to visualize the DNA using a confocal microscope.

#### Gel Retardation Assay

The binding of Sarconesin to *E. coli* DH5α genomic DNA was evaluated by a gel retardation assay (Teng et al., 2014). *E. coli* genomic DNA was extracted following the method of Landry et al. (1993). Seven two-fold increasing amounts of Sarconesin peptide (3.1 to 200 μM) were incubated for 1 hour with 500 ng of genomic DNA. The mixture was incubated for 1 hour at room temperature and analyzed by electrophoresis on a 0.8% agarose gel (Yang et al., 2017).

#### Statistical Analysis

All statistical analyses were performed using GraphPad Prism software (version 7.00). Bacterial growth curve after Sarconesin treatment was compared to the untreated control using a one-way ANOVA (α = 0.05). Statistical comparison of combination treatment in toxicity assays was done using a one-way ANOVA (α = 0.05) with Dunnett’s multiple comparisons test.

## Results

### Peptide Purification

**FIGURE 1:**
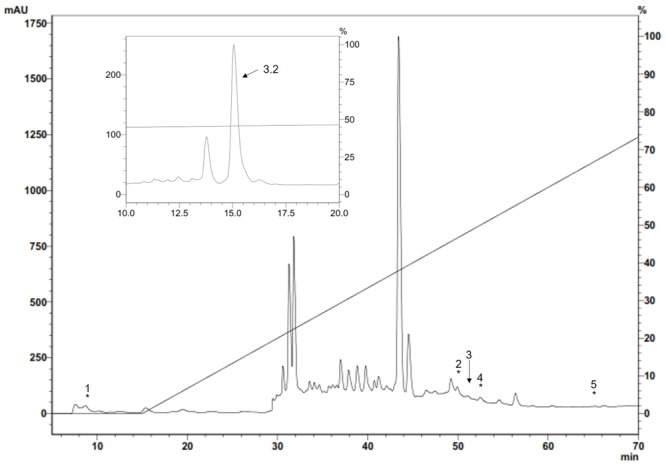
Antimicrobial fractions from *S. magellanica* ES eluted with 80% ACN from a Sep-Pak cartridge obtained from the first purification step by RP-HPLC. Chromatography involved using a semi-preparative Jupiter C18 column (10 μm; 300A; 10mm × 250mm) with a 0–80% linear gradient of ACN/0.05% TFA in H2O/0.05% TFA for 60 min at 2 mL/min flow rate. Absorbance was monitored at 225 nm. The fractions indicated by an asterisk had antimicrobial activity and were eluted at 8.1, 50.9, 51.7, 52.1, and 64.9 min; fraction 3 labeled with an arrow was chromatographed again in the same system with an analytic Jupiter C18 column (10um; 300A; 4.6mm × 250mm) and run in 44–54% solution B for 60 min (inset). The eluted 3.2 fraction (i.e., Sarconesin) had antibacterial activity and was purified.

ES material, analyzed by RP-HPLC, was lyophilised, suspended in water and antibacterial activity was tested. Antibacterial activity was quantified by plate growth inhibition assay using a Gram-positive *M. luteus* A270 bacteria as test-organism (**Figure 1**). Five of these fractions had antibacterial activity. Fractions 2, 3, 4, and 5 had anti-*P. aeruginosa* activity whilst the other compounds having no activity against *P. aeruginosa* were tested against the Gram-positive *M. luteus*. Activity was found in fraction 1; fractions having antimicrobial activity were eluted at 8.1, 50.9, 51.7, 52.1, and 64.9 min and chromatographed again in the same system with an analytic C18 column. All antimicrobial fractions were analyzed by mass spectrometry; when compared through a preliminary database search, just fraction 3 showed homogeneity with Diptera proteins. Purification of this fraction revealed the 3.2 molecule, having antibacterial activity against *M. luteus*.

### Antimicrobial Assays

**FIGURE 2:**
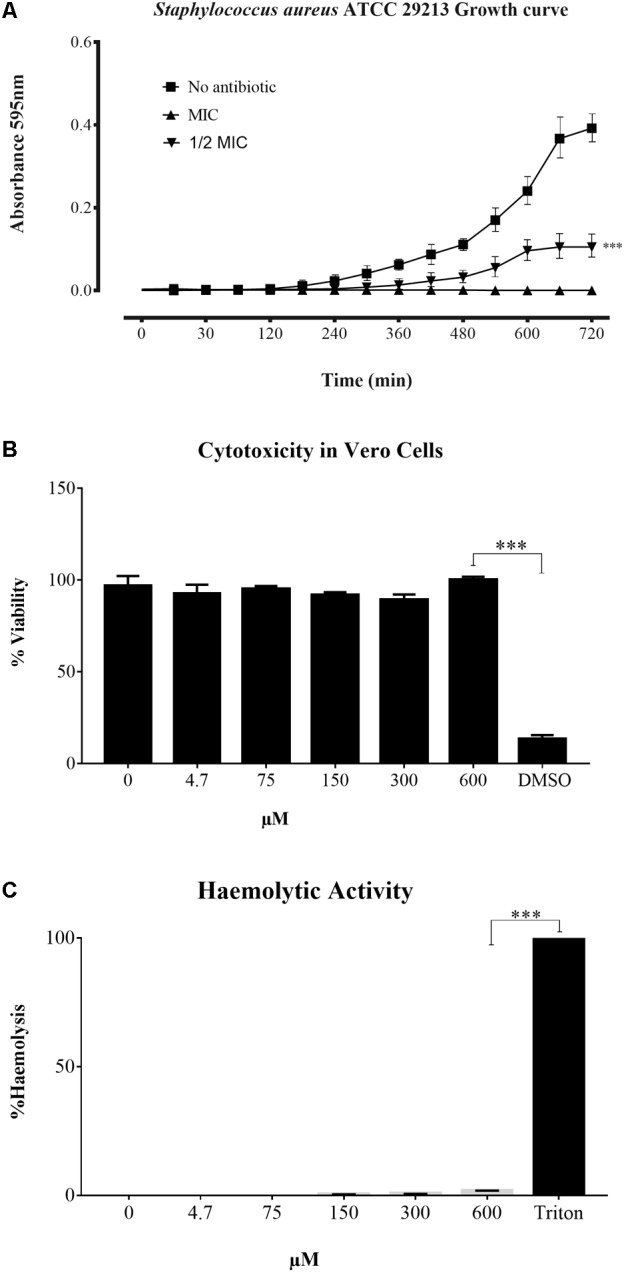
Time growth curve and toxicity assays. **(A)** Growth curve of *S. aureus* ATCC 29213 incubated with Sarconesin. Bacterial growth was inhibited and antibacterial effect was detected in the exponential phase. **(B)** Cytotoxicity and **(C)** Haemolytic activity of Sarconesin on the VERO cell line and fresh human red blood cells respectively, showing a very low toxicity even at the maximum tested concentration of 600 μM.

The peptides were studied regarding their potential for inhibiting Gram-positive and Gram-negative bacterial growth. Sarconesin MIC was the same (4.7 μM) for *M. luteus* A270 and *P. aeruginosa* ATCC 27853; minimum MIC (1.2 μM) was obtained for *S. aureus* ATCC 29213 and *S. epidermidis* ATCC 12228, and *E. coli* D31 and DH5α MIC was 2.4 μM (**Table 1**). Sarconesin MIC exhibited its effect in the exponential phase of *S. aureus* growth curve, which was reached after more than 180 min, incubation with ½ MIC showed a decrease of the bacterial growth (**Figure 2A**).

**Table 1:** MIC, minimum inhibitory concentration; MIC refers to the concentration needed for achieving 100% inhibition of growth.

Strain		Sarconesin MIC
Gram +	*M. luteus* A270	4.7 μM
	*S. aureus* ATCC 29213	1.2 μM
	*S. epidermidis* ATCC 12228	1.2 μM
Gram -	*P. aeruginosa* ATCC 27853	4.7 μM
	*E. coli* D31	2.4 μM
	*E. coli* DH5α	2.4 μM
	*S. enterica* ATCC 13314	2.4 μM

### Toxicity

The CC activity of Sarconesin was tested against the Vero cell line (**Figure 2B**). No sign of CC was observed with Sarconesin, even at the highest tested concentration, i.e., 600 μM. The viability of the cells was approximately 92% after exposure to Sarconesin. Selectivity index was not calculated as no CC50 values were found in the maximum evaluated concentrations. A very low (< 2%) haemolytic activity was observed upon incubating human red blood cells with Sarconesin at the highest concentration tested (600 μM) (**Figure 2C**).

### Mass Spectrometry

**FIGURE 3:**
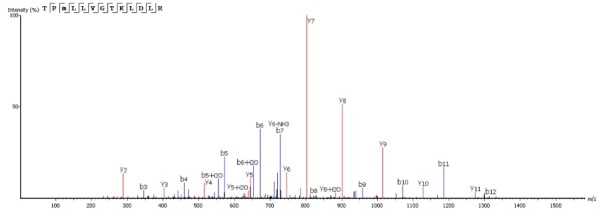
The complete Sarconesin aa sequence was obtained by mass spectrometry (MS/MS) fragmentation; representative *de novo* sequencing of Sarconesin. CID spectrum from mass/charge (m/z) of its double charged ion gave [M + 2H]2 + , m/z 736.9266. The ions from y (red) and b (blue) series (marked at the top of the spectrum) represent the primary structure: TPm( + 16)LLVGTKLDLR. The sequenced peptide’s internal fragments whose ions were found in the spectrum are represented by standard aa letter code.

Mass spectrometry analysis of the Sarconesin fraction revealed a molecule having a mass of 1,471.84 Da. The complete Sarconesin aa sequence obtained by PEAKS *de novo* sequencing revealed a 13 aa sequence having a post-translational modification (PTM): TPm( + 16)LLVGTKLDLR. Collision-induced dissociation spectrum from mass/charge (m/z) of its double charged ion gave [M + 2H]2 + ,m/z 736.9266 (**Figure 3**). Characterizing the peptide’s primary structure with the MASCOT tool gave the TPFLLVGTQIDLR sequence.

Knowing the sequence, proteomic, and peptidomic bioinformatics tools were used for predicting Sarconesin’s significant physicochemical characteristics. ExPASy’s (SIB Bioinformatics Resource Portal) PepDraw and Pep-Calc.com sequence analysis yielded a potential peptide isoelectric point (pI), molar extinction coefficient and net charge (**Table 2**). The peptide was predicted to have one Asp negatively-charged aa residue and a positively-charged Arg residue, thereby contributing to the peptide’s neutral characteristics (0 net charge). Four of the 13 aa were predicted to be hydrophobic (1 Ile, 3 Leu), suggesting poor water solubility for Sarconesin. ExPASy’s ProtParam tool predicted that the peptide would remain intact for up to 7.2 h in mammalian reticulocytes (*in vivo*), >20 h in yeast and >10 h in the Gram-negative bacterium *E. coli* (*in vivo*). This was likely due to the presence of a Thr (T) residue at the N-terminus.

**Table 2:** Physicochemical parameters calculated using ExPASy PepDraw and Pep-Calc.com (accessed April 30th 2018).

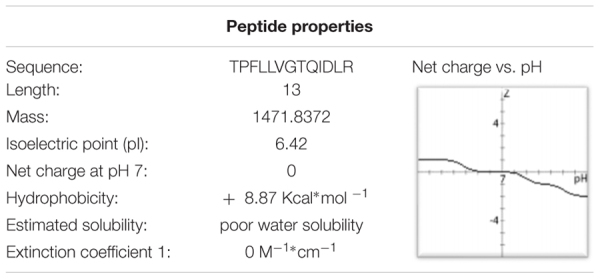

### Protein Model

A search for TPFLLVGTQIDLR in databases found matches with cell division control protein 42 (CDC42) sequences from humans, cows and fruit flies, having 100% sequence similarity. All sequences are referred to by their NCBI accession numbers^6^ to minimize confusion: CDC42 cell division control protein 42 homologue OS = *Bos taurus* (Q2KJ93), CDC42_HUMAN Chain A, structure of the Rho Family Gtp-binding protein Cdc42 in complex with the multifunctional regulator Rhogdi (gi| 7245832| 1DOA_A), CDC42_DROME CDC42 homologue OS = *Drosophila melanogaster* GN = Cdc42 PE = 1 SV = 1 (P40793). The BLASTp 2.6.1 + tool for comparing the sequence obtained with those for other *Lucilia* species proteins found 69% identity with a similar sequence previously report as a Ras-related protein Rac1 [*Lucilia cuprina*, another blowfly from the Calliphoridae family]: GenBank: KNC23156.1.

**FIGURE 4:**
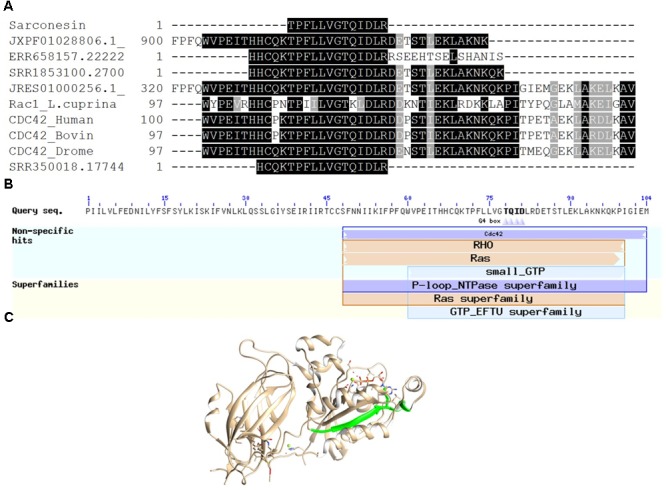
Sarconesin alignment and protein model. **(A)** Sarconesin multiple sequence alignment against selected proteins. (https://www.ncbi.nlm.nih.gov/): CDC42 cell division control protein 42 homologue OS = *Bos taurus* (Q2KJ93), CDC42_HUMAN Chain A, structure of the Rho family Gtp-binding protein Cdc42 in complex with the multifunctional regulator Rhogdi (gi| 7245832| 1DOA_A), CDC42_DROME Cdc42 homologue OS = *Drosophila melanogaster* GN = Cdc42 PE = 1 SV = 1 (P40793), Ras-related protein Rac1 [*Lucilia cuprina*] GenBank: KNC23156.1. Sarconesin has 100% sequence similarity with CDC proteins and 69% with Rac from *L. cuprina*. Translated sequences from *L. sericata*. Genome scaffold (JXPF01028806.1), transcriptomes ERR658157.22222021.1 and pupa SRR350018.17744834.1 genome scaffold (JRES01000256.1) and *L. cuprina* transcriptome SRR1853100.27006533.2. **(B)** Conserved domains found in JXPF01028806.1 *L. sericata* Blastp, showing Sarconesin a conserved residue from CDC42 domain. **(C)** Representative model of human CDC42 (PDB ID: 1DOA_A). Sarconesin is encrypted in a site between residues 111 and 123 (green), which folds as a β-sheet.

Sarconesin sequence was sought in the genomes and transcriptomes reported for *L. cuprina* (genome ASM118794V1, transcriptome SRX907163) and *L. sericata* (genome ASM101483V1, transcriptome ERX614478, 3-4 day pupa transcriptome SRX087348). Sarconesin was found in all of them (*L. sericata* genome scaffold JXPF01028806.1 and transcriptomes ERR658157.22222021.1 and pupa SRR350018.17744834.1. *L. cuprina* genome scaffold JRES01000256.1 and transcriptome SRR1853100.27006533.2 (accessed May 16th, 2018) (**Figure 4A**).

The exon containing Sarconesin in the JXPF01028806.1 scaffold (GenBank) was located and compared to other proteins by Blastp for determining which organisms had the greatest similarity with the gene. It was shown that this gene was mainly present in other Diptera species (*Stomoxys calcitrans* XP_013103099.1, *Drosophila sechellia* XP_002039460.1, *Musca domestica* XP_005189222.1, *Anopheles gambiae* CAA93820.1, and *Ceratitis capitate* XP_004518385.1), having 100% similarity. It was established that Sarconesin formed part of a CDC42 conserved domain (**Figure 4B**). The Sarconesin model was built using CDC42’s known structure (PDB ID: 1DOA) since it has 100% identity with bovine CDC42 and a PDB model is available. **Figure 4C** shows the homology model constructed for Sarconesin.

### Circular Dichroism (CD)

**FIGURE 5:**
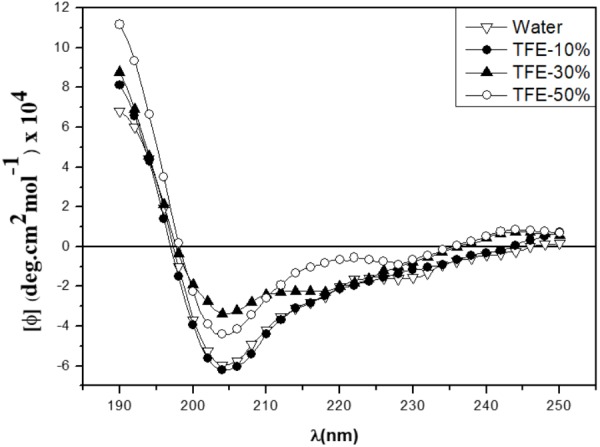
CD spectra of the Sarconesin peptide in water and different TFE/Water proportions.

CD deconvolution software was not suitable for peptide analysis, since it was designed for larger proteins (Bochicchio and Tamburro, 2002), so the peptide’s secondary structure analysis was thus made in a qualitative way, by comparing with CD spectra obtained from known secondary structures of the literature. CD spectra of the peptide were obtained at 25°C, in water and in TFE/water ranging from 0 to 50% v/v (**Figure 5**). In water, the CD spectrum showed a strong negative band around 208 nm and a moderate positive band around 190 nm. As TFE concentration increased, the negative band became less intense and the positive band became more intense and a shoulder between 220 and 230 nm appeared. TFE has the property of aggregating itself around peptide molecules promoting the displacement of the solvation layer, thereby favoring the formation of intra-peptide hydrogen bonds, stabilizing the peptide’s secondary-structure. CD spectra features suggested a mixture of 3_10_-Helix and α-Helix conformations (Berova et al., 2012). In water, the 3_10_-Helix proportion was favored and as the TFE concentration in solution increased, the α-Helix contribution also increased, suggesting that the α-Helix conformation could be predominant in low dielectric environment, like in a phospholipidic membrane.

### Mechanism of Action

#### Membrane Integrity and Esterase Activity

**FIGURE 6:**
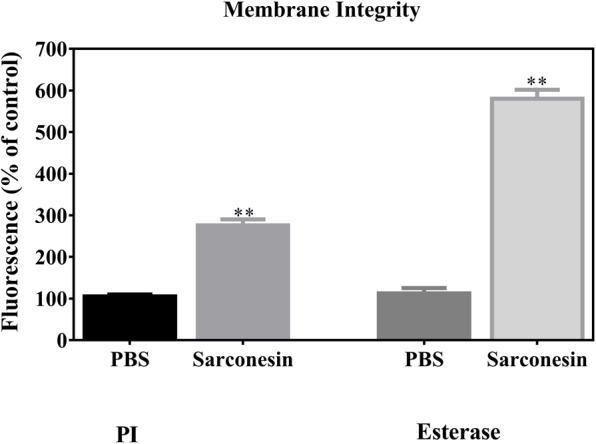
Representative image showing change in the mean fluorescence intensity of fluorescence probes PI and CFDA (esterase activity) in *E. coli* bacteria. Histogram represents changes in the mean ± S.D of PI and CFDA fluorescence, obtained from three independent experiments (^∗∗^*p* < 0.05 vs. control).

**FIGURE 7:**
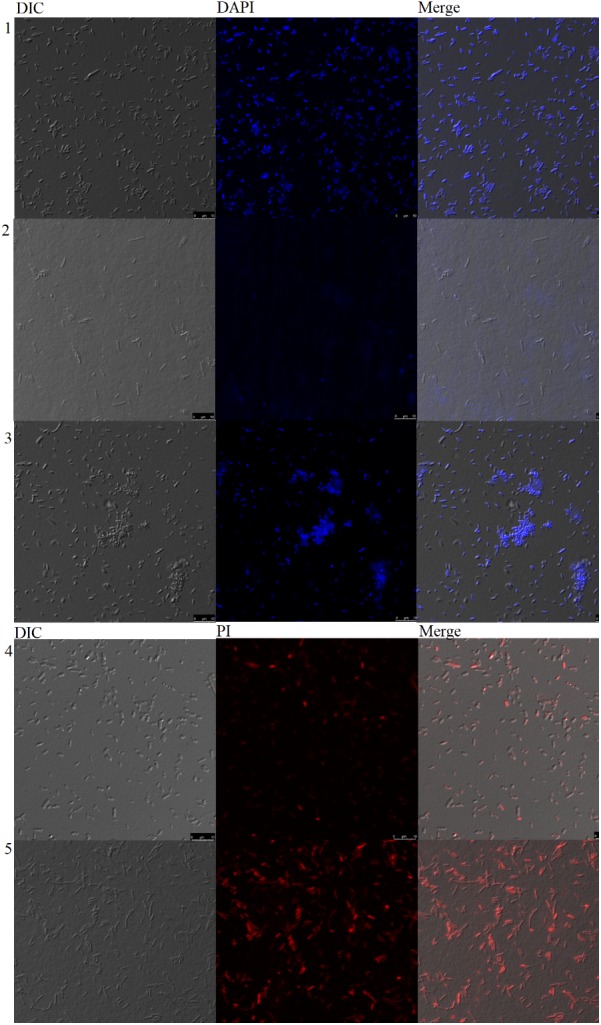
Confocal microscopy analysis of bacteria incubated with Sarconesin. PBS-, Ciprofloxacin- and Sarconesin-treated bacteria stained with DAPI (1-3). PBS- and Sarconesin-treated bacteria stained with PI (4–5).

The red fluorescent dye propidium iodide (PI), which is kept on the outside of intact membranes, can penetrate damaged cell membranes and intercalate into nucleic acids. The fluorescence intensity of PI indicates the level of cell membrane integrity. In the absence of peptide, cells exhibited no PI staining, indicating that membranes were intact (**Figures 6, 7**). After treatment with Sarconesin, the percentage of PI-permeable *E. coli* cells increased. This suggested that the inner membrane of *E. coli* was disrupted after treatment with Sarconesin. Also, an alteration in the esterase activity when compared with bacteria control was observed.

#### DNA Staining

Neither untreated bacteria nor Sarconesin-treated bacteria showed DNA fluorescence, indicating that DNA was not denatured with Sarconesin treatment (**Figure 7**).

#### DNA Gel Movement Retardation

**FIGURE 8:**
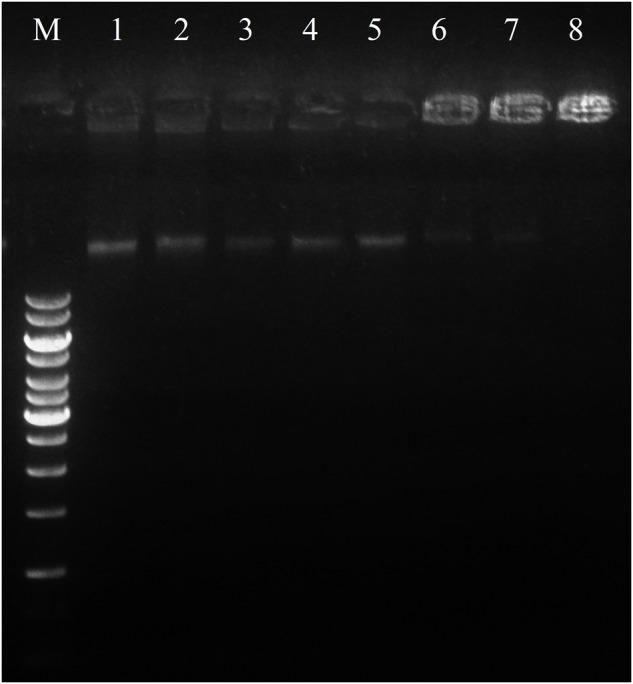
Interaction of Sarconesin with bacterial genomic DNA by a gel migration assay. M, DNA marker GeneRuler 1Kb; 1–8, the Sarconesin concentrations were 0, 3.1, 6.25, 12.5, 25, 50, 100, and 200 μM.

In an attempt to clarify the molecular mechanism of action, DNA-binding properties of Sarconesin were examined by analysing electrophoretic migration of DNA. The migration of *E. coli* genomic DNA suppressed by Sarconesin at 50, 100, and 200 μM (**Figure 8**). This result indicated that Sarconesin can bind to bacterial DNA.

## Discussion

Bacterial resistance against antibiotics has created special interest in searching for new compounds as potential antimicrobial drugs which might be more effective in developing new therapeutic tools (Chernysh et al., 2015). The present work led to finding a new sequence from *S. magellanica*; its antibacterial activity was screened and its biochemical and structural properties were elucidated by sequence homology. One AMP responsible for the antibacterial activity previously reported in *S. magellanica* was found (Diaz-Roa et al., 2014). Sarconesin was seen to have 1,471.8372 Da mass and similarity with Cdc42 and Rac proteins; the AMP was embedded in a site between human Cdc42 residues 111 and 123, folding as a β-sheet. A search for the peptide in AMPs database did not reveal any similarity with previously reported AMPs; however, this new peptide could be part of the family of linear AMPs (Giuliani et al., 2006).

The MIC obtained for Sarconesin in this study suggested a potent activity, similar to that previously reported for other peptides that are active below a 32 μg/mL concentration (Cézard et al., 2011), also, the thanatin peptide has shown a MIC below 2.5mM, which highlights that Sarconesin required less peptide to inhibit bacterial growth (Bulet et al., 1999). This further supports the importance of new effective substances, knowing that several bacteria do become resistant after some days or even hours of exposure (Giacometti et al., 1999). Also, the growth curve of *S. aureus* showed that Sarconesin has effect in less than 180 min of incubation. It should be stressed that the fractions having antibacterial activity were absent in the peaks having the greatest absorbance; this has already been observed in other work where defensin, diptericin (Chernysh et al., 2015) and lucifensin have been detected in very tiny peaks (Cerovsky et al., 2010).

Sarconesin has C-terminal R and N-terminal T residues, when a search by homology was made, a K residue was found immediately before the N-terminal T, suggesting that it might be targeted by trypsin-like activity (Chay Pak Ting et al., 2011). The peptide obtained could have resulted from the presence of some proteases and other enzymes in the ES, taking into account that our experimental procedure did not involve trypsin treatment (Nigam et al., 2010); it has already been reported that ES have trypsin and chymotrypsin in their content (Sandeman et al., 1990;Telford et al., 2011). Sarconesin could be a product of processing the Cdc42 or Rac protein and have other functions in the blowfly related to cell cycle; the derived Sarconesin also has antibacterial activity. It is worth emphasizing that Rac’s antimicrobial activity has not been reported before in Calliphoridae blowflies.

As Sarconesin was also present in *S. scrofa* and the flies’ food supplement was liver, it could be possible to assume that the peptide was a subproduct of CDC42 from *S. scrofa* and not from *S. magellanica*. However, the presence of Sarconesin in the transcriptome and genome of other *Lucilia* species showed that this peptide is present in such blowflies, maybe as a sub-product of Diptera. Indeed, multi-omics studies of maggots for larval therapy usually involve using insects fed on bovine liver (Sze et al., 2012; Anstead et al., 2015). The Sarconesin peptide was also present in studies with maggots fed on sheep blood agar as supplement (Poppel et al., 2015; Franta et al., 2016). This exon was also searched in through blast to discard whether it had greater similarity with Diptera species than with *S. scrofa*. It was found to be more similar with Diptera species having different feeding habits and was also associated with a CDC42 conserved domain. Sarconesin was also found in pupa transcriptome having no contact with liver residue, showing that this peptide’s origin could most likely be from the fly.

ES pH is usually 8–8.5 (i.e., in *Phaenicia sericata*) (Erdmann, 1987; Thomas S. et al., 1999) because of a waste product (ammonia), since ammonia increases wound pH, resulting in alkaline conditions which are unfavorable for many bacterial species (van der Plas et al., 2008; Cazander et al., 2009). Sarconesin’s net charge would thus be negative as Sarconesin is present in ES and knowing that a protein’s net charge is positive at pH below pI (Shaw et al., 2001) (**Table 2**). This makes Sarconesin an anionic peptide in normal conditions in ES. The mechanism of action regarding bacteria could involve translocation across the membrane (the common mode of action for anionic peptides) (Phoenix et al., 2013), knowing that AMPs can function as direct antimicrobial compounds (Diamond et al., 2009) and also as effector molecules induced upon microbial infection (Zhang et al., 2013).

Sarconesin has 100% sequence similarity with different organisms’ CDC42 protein and 69% identity with RAC from *L. cuprina*. Both proteins form part of the Rho-family GTPases (Wennerberg and Der, 2004); Sarconesin may have a similar intracellular mechanism of action, knowing that this protein’s expression activates growth factors (Higuchi et al., 2001) and acts as a molecular switch by responding to exogenous and/or endogenous signals, relaying such signals to activate an intracellular biological pathway’s downstream components (Johnson, 1999). As the PI assay showed, Sarconesin was confirmed to have a membrane disruption mechanism that has already been reported for other AMPs due to electrostatic interactions, which can be probably followed by hydrophobic patches’ insertion into the non-polar interior of the membrane bilayer (Khamis et al., 2015), having a barrel-stave model for the channel pore similar to Alamethicin that, as Sarconesin, has a 3_10_-helix conformation (Nagao et al., 2015). Some morphological changes on bacteria were observed in Sarconesin PI experiment (**Figure 7**), these most likely occurred when the antimicrobial agent attacked the cell membrane, as has been previously reported by Hyde (Hyde et al., 2006).

Sarconesin induced the release of 6-carboxyfluorescein, indicating an effect resulting from a transient destabilization of the bilayer upon initial interaction, a similar effect previously reported for the magainin-2 peptide (Oren and Shai, 1998). The bacterial DNA, when incubated with Sarconesin and DAPI, did not show degradation and the gel retardation assay showed that Sarconesin strongly bound to DNA *in vitro*, thus suggesting the possibility of inhibiting intracellular functions via interference with DNA (Shi et al., 2016).

The selectivity index was not established, nevertheless, no CC was found. These findings would point toward the existence of an appreciable selectivity of this compound against bacteria and, therefore, this observation may be an indicator of their safety as drugs for mammalian organisms. To verify whether the peptide was able to disrupt human erythrocyte membranes in an attempt to evaluate the peptide’s future pharmacological potential, a haemolytic assay was performed, where the peptide displayed a very low (<2%) haemolytic activity in a 600 μM final concentration. The haemolytic activity decreased to 0% in a concentration ranging between 600 and 300 μM (**Figure 2C**). As expected, the peptide has no relevant toxic potential, even when tested a concentration 128 times higher than the *M. luteus* and *P. aeruginosa* MIC (i.e., 4.7 μM), based on the fact that Sarconesin is identical with a conserved domain from CDC42, molecule present in different human cells that acts as a molecular switch in the control of a variety of eukaryotic processes (Johnson, 1999).

Sarconesin may also have implications for wound healing. It has previously been reported in cell culture that ES from other necrophagous flies increase fibroblast proliferation for wound healing (Polakovicova et al., 2015). Also, specifically Sarconesin has been previously reported as an angiogenesis biomarker of recovery after acute kidney injury, so could be a good candidate for future wound evaluation activities (Titulaer, 2013). The Rho family also has wound-healing properties and the role of GTPases in epithelial remodeling during wound-healing and epithelial-mesenchymal transitions has also been previously reported (Van Aelst and Symons, 2002). There is also evidence that Cdc42 plays a major role in wound healing, regarding host defense against infection (Lee et al., 2013). Previously identified natural AMPs from insects are produced by bacteria, fungi, numerous invertebrates, vertebrates, and plants and are usually associated with killing microbes, although they could also be involved in wound repair, inflammation, chemotaxis, and cytokine activity (Ratcliffe et al., 2014).

This article reports, for the first time, a small antimicrobial peptide which is a member of a new Rho family; it contains 13 residues and is active against Gram-positive and Gram-negative bacteria. The native peptide was purified from *S. magellanica* by RP-HPLC and characterized by amino acid sequencing. Further studies aimed at evaluating its activity against other bacteria, fungi, viruses, and parasites are needed, as well as ascertaining its mechanism of action and investigating its action in wound healing.

## Data Availability

The datasets analyzed during the current study are available from the corresponding author on reasonable request.

## Author Contributions

AD-R carried out the design, conduct of the study, and wrote the manuscript. FB, MP, and PJ contributed to the idea, revised the manuscript and helped data interpretation and supervised the work. All authors read and approved the final manuscript.

## Conflict of Interest Statement

The authors declare that the research was conducted in the absence of any commercial or financial relationships that could be construed as a potential conflict of interest.

## Footnotes

1 http://aps.unmc.edu/AP/main.php

2 http://blast.ncbi.nlm.nih.gov/ [accessed April 23, 2018]

3 http://pepcalc.com/ [accessed April 30, 2018]

4 http://www.ch.embnet.org/software/BOX_form.html

5 http://www.ebi.ac.uk/thornton-srv/databases/profunc/ [accessed April 30, 2018]

6 https://www.ncbi.nlm.nih.gov/
